# A Rare Non-smoking-Related Spindle Cell Carcinoma of the Lung: A Case Report

**DOI:** 10.7759/cureus.78151

**Published:** 2025-01-28

**Authors:** Luisa Fernanda Gomez Garcia, Shamill Morel, Manuel Alvarado McDougal

**Affiliations:** 1 School of Medicine, Pontificia Universidad Catolica Madre y Maestra, Santiago de los Caballeros, DOM; 2 General Medicine, Instituto Oncologico Regional del Cibao, Santiago de los Caballeros, DOM; 3 Oncology, Instituto Oncologico Regional del Cibao, Santiago de los Caballeros, DOM

**Keywords:** cancer, chemotherapy, clinical case report, lung tumor, spindle cell carcinoma

## Abstract

This paper introduces the case of a 30-year-old female diagnosed with pulmonary spindle-cell carcinoma (PSCC), a rare non-small cell lung carcinoma, with no history of tobacco use or any other known risk factors. The patient, with a diagnosis of neurofibromatosis type 1, showed symptoms of night fevers, thoracic pains, and massive pleural effusion, which led to a series of diagnostic studies, including imaging studies, biopsies, and immunohistochemistry analyses. The results from the pulmonary biopsy were used to make the definitive diagnosis of PSCC with negative results for the typical markers for this type of cancer. Following the diagnosis, the patient started a chemotherapy regimen consisting of doxorubicin and ifosfamide, with which she has survived for more than six months, despite the poor prognosis associated with PSCC. This case report highlights the rarity of this histological subtype, the difficulty in its diagnosis and management, the importance of completing a comprehensive diagnostic approach, and the need for further studies to improve the prognosis of these patients.

## Introduction

Pulmonary spindle cell carcinoma (PSCC) is a very aggressive non-small cell lung cancer (NSCLC), which, although rare, represents around 0.1%-0.4% of all lung cancers. In the broader context of lung cancer, NSCLC accounts for 85% of cases, with adenocarcinoma being the most common subtype, while small cell lung cancer (SCLC) makes up only 15% [1]. PSCC belongs to the subclassification of pulmonary sarcomatoid carcinoma, alongside other histological subtypes, such as giant cell carcinoma, pleomorphic pulmonary carcinoma, carcinosarcoma, and pulmonary blastoma [2]. This cancer has a poor prognosis, and the available literature, though limited, suggests that it typically presents at the lung’s periphery [3]. Immunohistochemistry analysis often results positive for pan-keratin, though this is not always the case [4].

A study published by Ung et al. in 2017 reported the considered relevant demographic data, where 77% of the patients diagnosed with pulmonary sarcomatoid carcinoma were men, with a mean age of 63 years old, and 87% presented previous history of smoking; the most common subtype being pleomorphic pulmonary carcinoma [5]. It is of interest to the authors to highlight that the clinical case presented involves a young adult woman with no history of smoking and negative immunohistochemistry results for pan-keratin and other markers, differing greatly from the existing statistics, being this peculiarity that calls for attention. This case report aims to serve as a contribution to the limited knowledge and literature on PSCC, focusing on the clinical course, the diagnostic challenges, and the management timeline/outcome and comparing its findings to the expected prognosis and management described in the literature.

## Case presentation

A 30-year-old Dominican woman, with a history of neurofibromatosis type 1 (NF1), presented to a health center in her community with a clinical profile characterized by non-measured fevers and diaphoresis of nocturnal predominance. This was accompanied by a two-day history of productive cough, shortness of breath, and pleural thoracic pain, considered 9 out of 10 on the subjective pain scale. The patient denied any smoking history, secondhand exposure, or occupational risk factors. The family history includes relevant findings of a brother with chronic kidney disease due to diabetes mellitus type 1 and aunts and uncles who suffer from breast cancer, cervix cancer, and unspecified leukemia.

**Figure 1:**
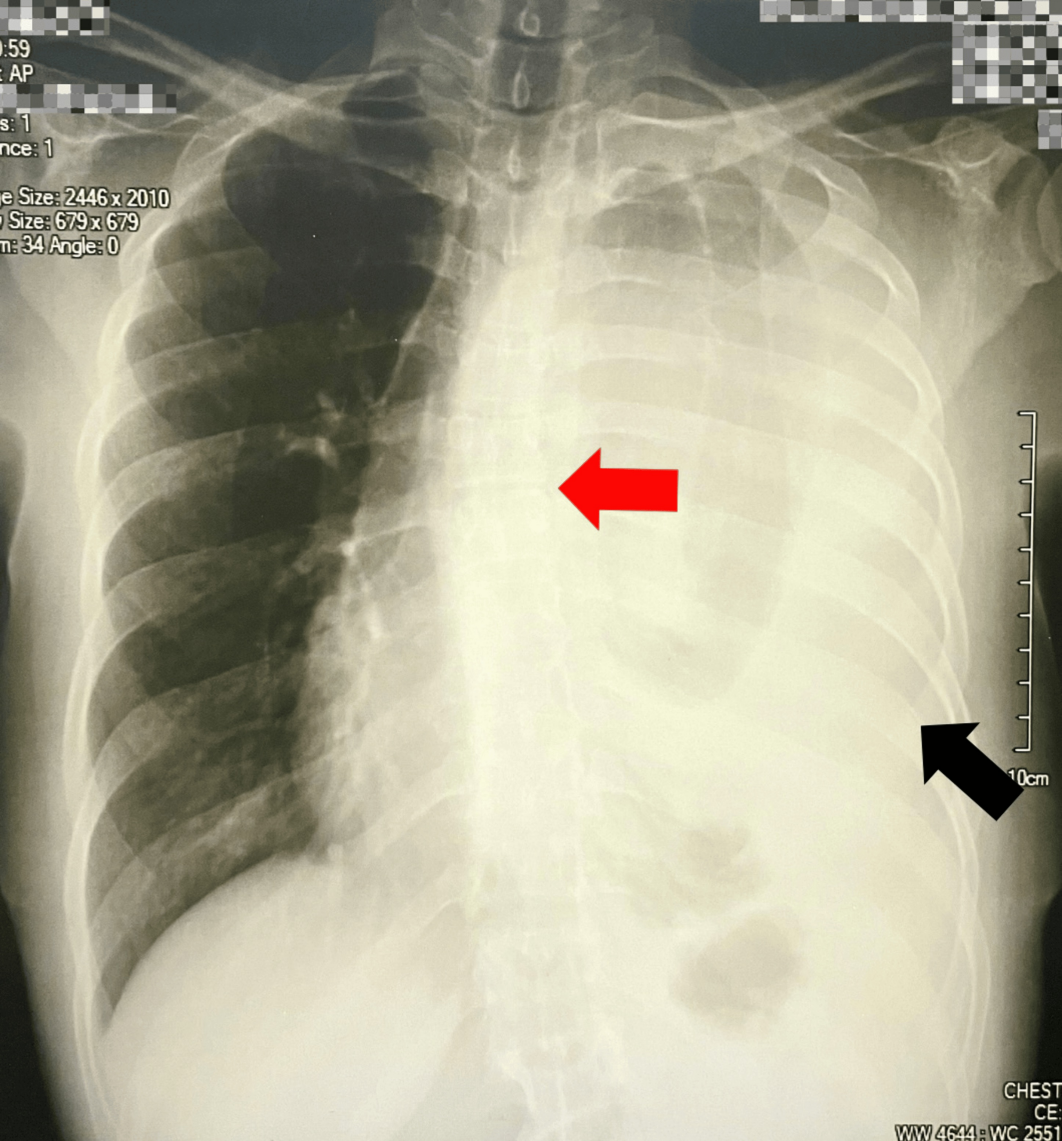
Chest X-ray The red arrow indicates the displacement of the structures towards the right hemithorax. The part indicated by the black arrow suggests what has been described as "massive pleural effusion".

Following chest X-ray imaging studies, which were indicative of a massive pleural effusion (Figure 1), the decision was made to perform an evacuative therapeutic thoracentesis with a 1,000 mL drainage with a turbid fluid (no cytology tests were run). Due to the limited resources to address the patient’s clinical condition, she was referred to a tertiary health center two days later, where she was admitted for 16 days for diagnostic and therapeutic purposes. After admission, imaging studies were repeated, and the pulmonology team initiated an empirical antibiotic treatment consisting of ceftriaxone and clindamycin. In addition, a pleural biopsy was ordered for further evaluation, reporting a moderate chronic inflammatory process with fibrotic changes. However, due to inconclusive results, it was decided to repeat the biopsy at an external institution.

**Figure 2:**
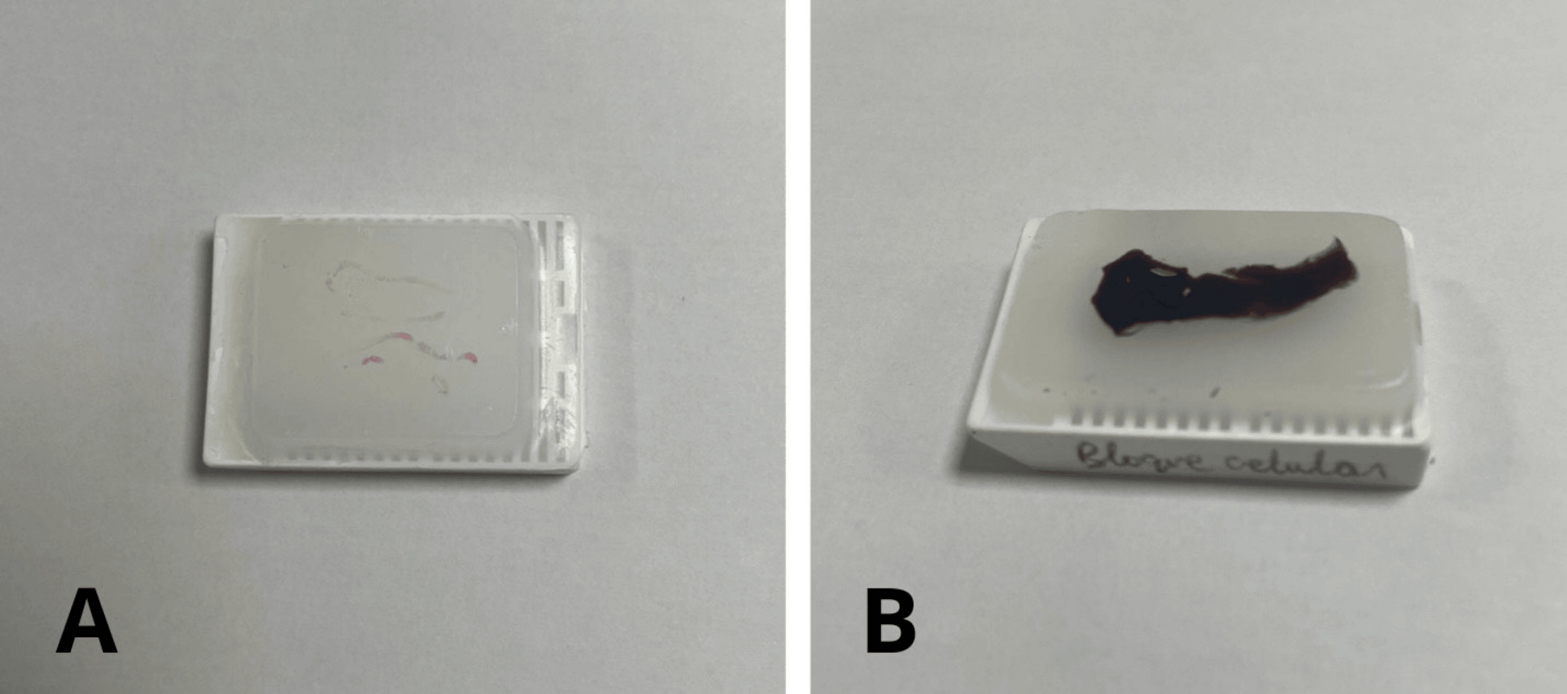
Histological blocks with tissue sections used for sample determination

**Figure 3:**
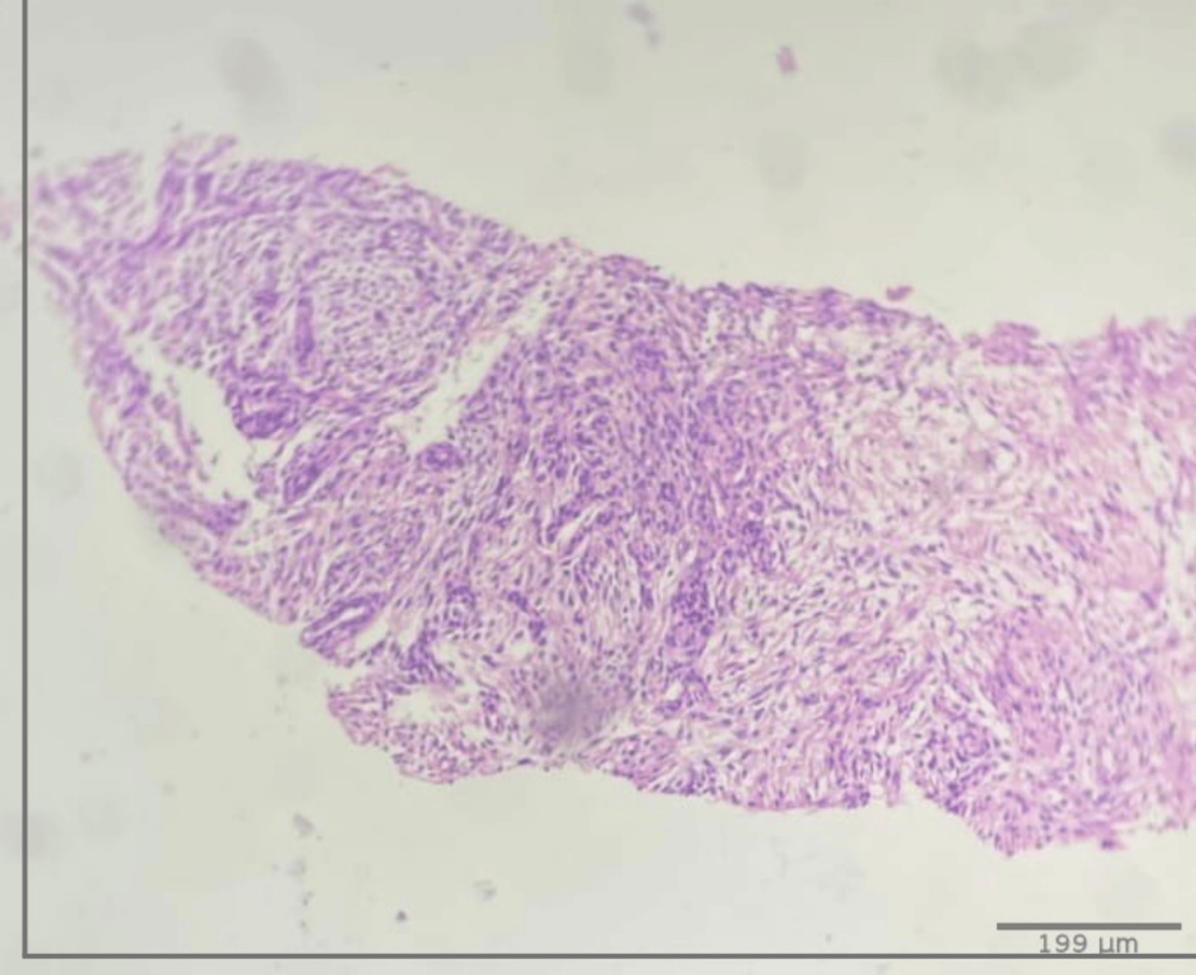
Microscopic tissue sample Lung biopsy tissue with hematoxylin and eosin (H&E) staining, at 4x microscopic magnification.

**Figure 4:**
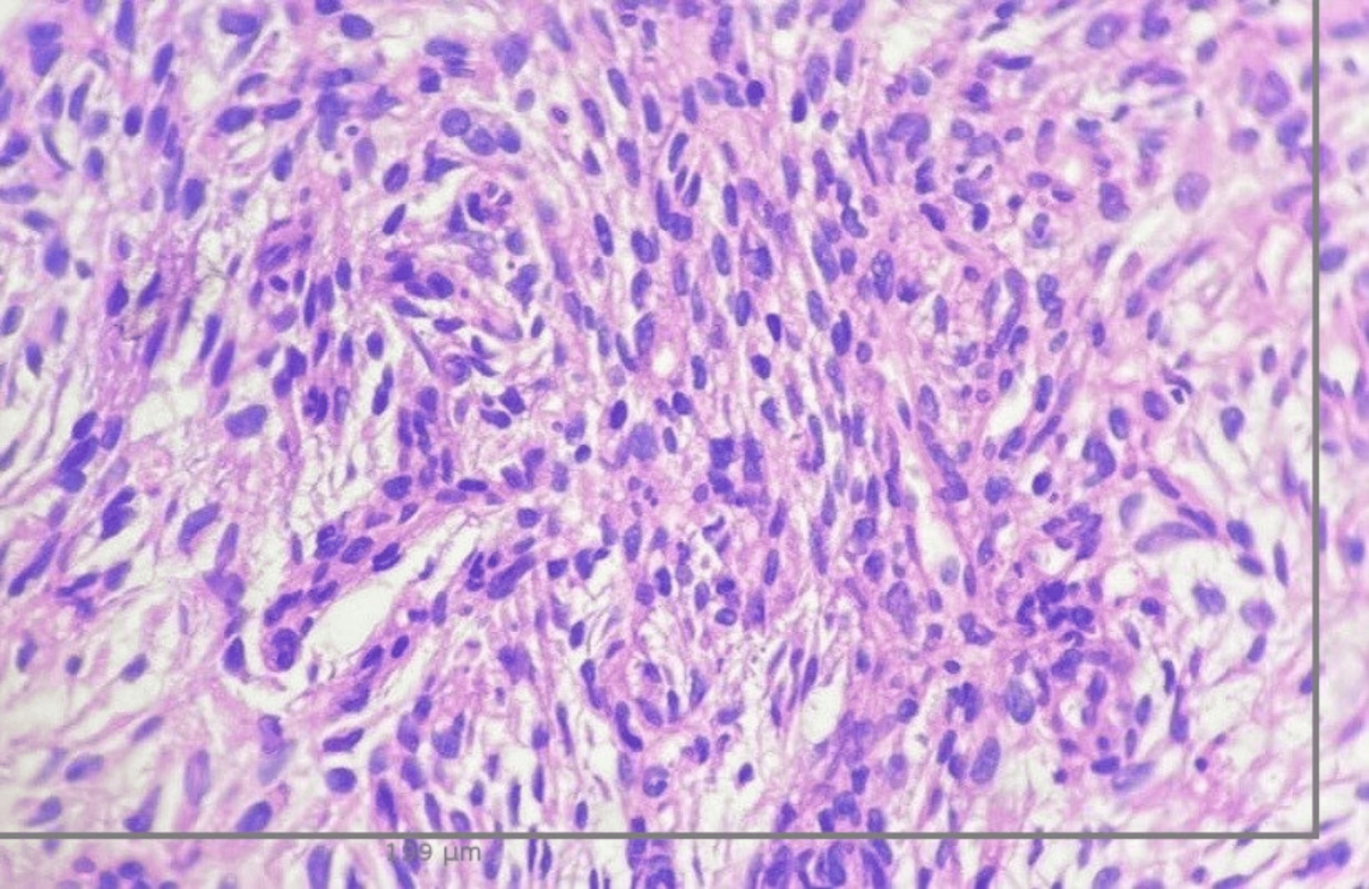
Microscopic tissue sample Lung biopsy tissue with H&E staining, at 40x microscopic magnification. The microscopic specimen stained with H&E shows irregular dense fibrous connective tissue infiltrated by neoplastic cells arranged in groups without a specific pattern. These cells in some areas are spindle-shaped with elongated nuclei, blunt edges, regular dense chromatin and scant eosinophilic cytoplasm. In other areas, the nuclei are ovoid with irregular edges, regular chromatin, and clear eosinophilic cytoplasm.

**Figure 5:**
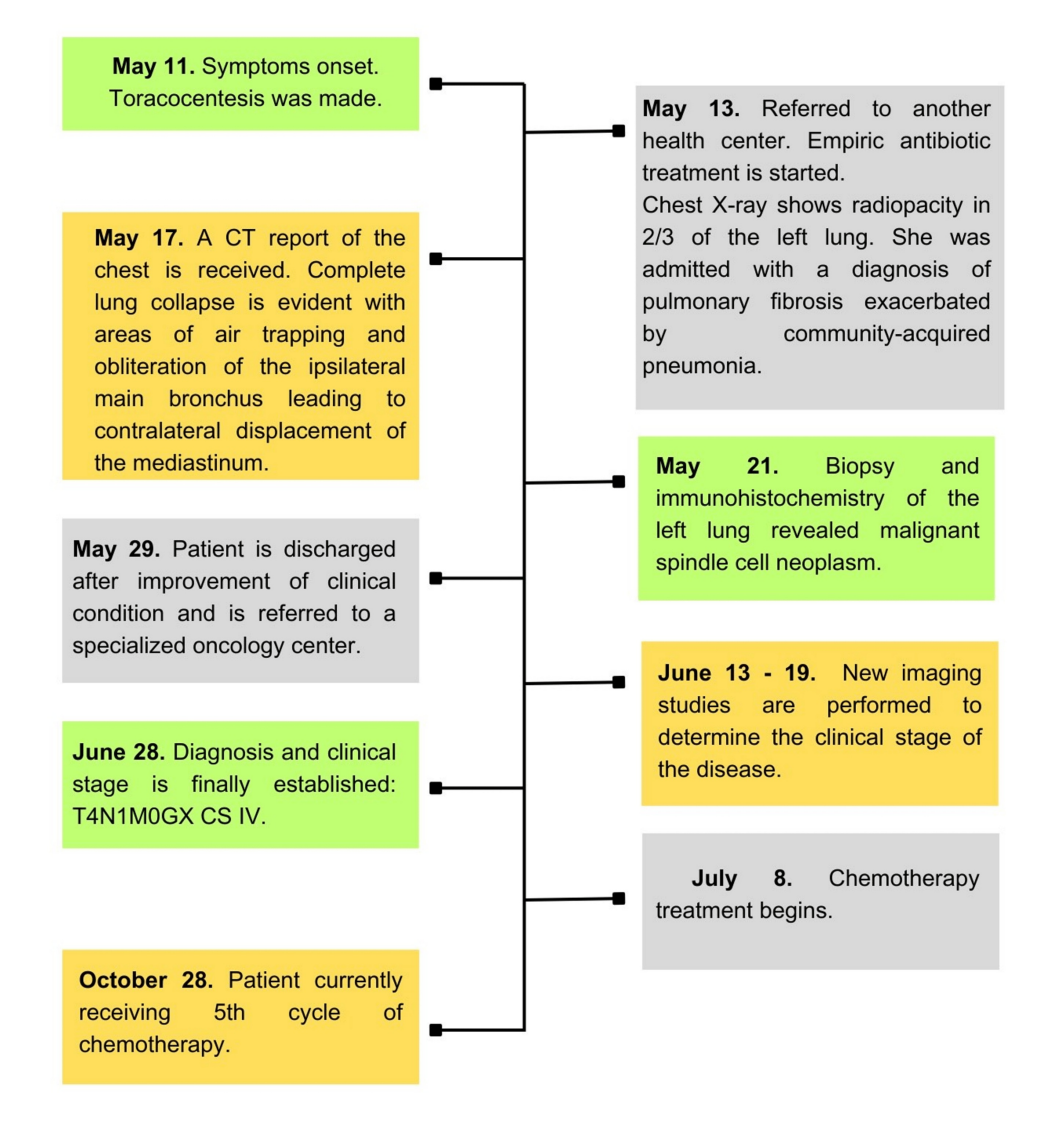
Timeline of interventions

After performing a new lesion biopsy in the upper left lung lobe guided by computed axial tomography (CAT), a mesenchymal spindle cell neoplasm was revealed both by the tru-cut technique and by fine needle aspiration (FNA) (see gross size section objects in Figures 2A, 2B). As part of the diagnostic protocol, immunohistochemistry (IHC) was performed on the samples obtained using the Benchmark GX system from Ventana/Roche, with negative results on all the requested markers (see Table 1). The microscopic sample showed irregular dense fibrous connective tissue exhibiting infiltration by neoplastic cells arranged in groups without a specific pattern. These cells in some areas are spindle-shaped with elongated nuclei, blunt edges, regular dense chromatin, and scarce eosinophilic cytoplasm. In other areas, the nuclei are ovoid with irregular edges, regular chromatin, and clear eosinophilic cytoplasm (Figures 3-4). This led to a diagnosis of malignant spindle cell neoplasm, and with the patient’s clinical condition improving, the pulmonology department discharged her, referring her to a specialized oncology center for outpatient care. A detailed timeline of interventions is depicted in Figure (5).

**Table 1:** Immunohistochemistry report Note: The negativity of the immunohistochemical markers CD45, S100, SOX-10, CD34, and calretinin helps exclude several types of tumors. The negativity of CD45, S100, SOX-10, and calretinin rules out hematologic, neuroectodermal (e.g., melanomas and schwannomas), and mesothelial tumors (e.g., mesothelioma). Additionally, the negativity of CD34 eliminates the possibility of vascular or hematopoietic tumors. These results contribute to confirming the diagnosis of spindle cell carcinoma of the lung (PSCC), even though pan-keratin would have been expected to be positive.

Immunohistochemical marker	Result
Pan-keratine AE1/AE3	Negative
CD45	Negative
S100	Negative
SOX-10	Negative
CD34	Negative
Calretinin	Negative

**Figure 6:**
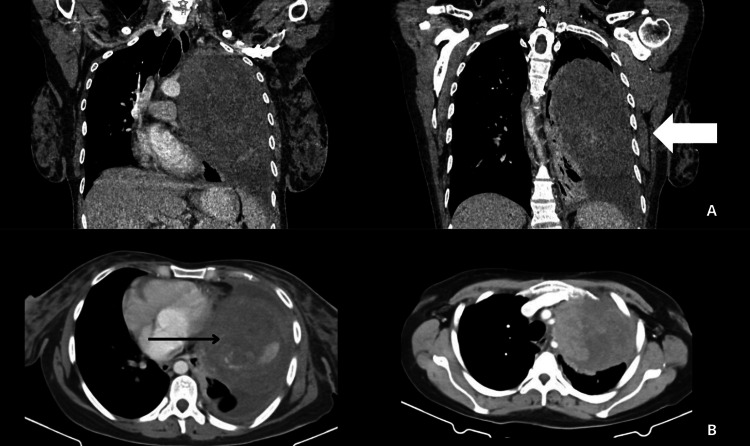
Computed tomography scan of the chest A: The white thick arrow shows the pulmonary mass lesion in a coronal section view. The lesion has an approximate longitudinal dimension of 16.8 cm. B: The black thin arrow shows the lesion in a cross section view. The lesion has an approximate dimension of 11.02 cm anteroposterior and 10.3 cm transverse. This is an expansive lesion of the left lung that infiltrates the visceral and parietal pleura, in close contact with the following structures: pericardium, subclavian artery, and ipsilateral aortic arch. The lesion exerts a mass effect with contralateral mediastinal displacement.

Upon receiving the patient in the medical oncology department, chest, abdomen, and pelvic CT scans with contrast, along with a bone scan, were requested to rule out metastasis. The chest CT scan showed a left pulmonary lesion infiltrating the parietal and visceral pleura, measuring 11.02 x 10.3 x 16.8 cm, in contact with the pericardium, ipsilateral subclavian artery, and aortic arch, with a mass effect (Figures 6A, 6B).

After thorough evaluation, including a positron emission tomography scan excluding metastasis, the diagnosis and staging were concluded as follows: left lung malignant spindle-cell neoplasm, T4N1M0GX, Stage IV unresectable. Therefore, an induction chemotherapy regimen was planned based on the AIM scheme, consisting of doxorubicin 25 mg/m^2^ days 1-3, ifosfamide 2,500 mg/m^2^ days 1-4, mesna 2,500 mg/m^2^ days 1-4, every three weeks for six cycles, along with granulocyte-colony stimulating factor (G-CSF).

Follow-Up

**Figure 7:**
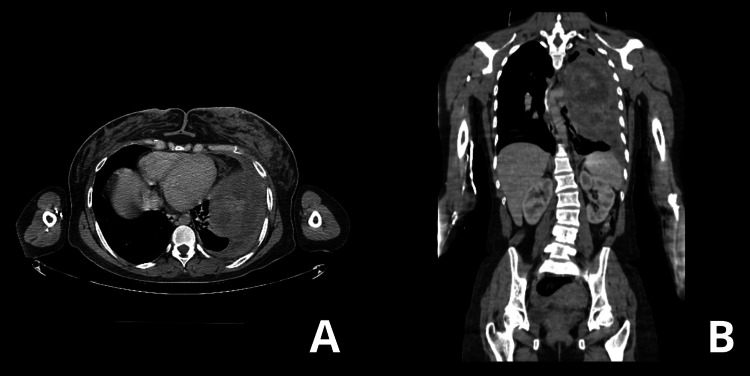
Follow-up computed tomography A, B: A known heterogeneous lesion is shown in the left lung, displacing the mediastinum to the right. The lesion has dimensions of 14.7 cm x 11.5 cm x 18.4 cm with mass effect.

With the onset of symptoms on May 11, 2024, the patient has completed five cycles of treatment to date. In other words, six months have passed since the initial presentation, and the patient remains alive and asymptomatic, thus exceeding the known median survival for PSCC, which is five months [6]. Prior to the completion of the established chemotherapy cycles, a control chest CT scan was performed to determine the status of the lesion. The result showed a lesion with minimal expansion in contrast to the initial CT scan, which shows a stable disease (Figures 7A, 7B).

## Discussion

Although there is no specific treatment for PSCC, it can be chosen based on the clinical stage of the case. The literature provides different treatment options that have been used for this type of cancer, including immunotherapy, targeted therapy, chemotherapy, surgery, radiotherapy, and traditional Chinese medicine. The latter is of particular interest in a case report with a 48-month survival post-treatment, compared to the known median survival of five months [6,7]. Since this patient’s case is Stage IV unresectable, the oncology department chose a chemotherapy regimen supported by a study published by Grobmyer et al., which showed a significant improvement in survival in patients with high-grade soft tissue sarcomas larger than 10 cm [8], consisting of doxorubicin, ifosfamide + mesna.

Diagnostic techniques such as immunohistochemistry, in most cases, help confirm the PSCC diagnosis. In 2016, Weissferdt et al. determined that PSCC typically stains for markers such as CAM5.2 in 93% of cases, CK7 or AE1/AE3 in 79%, and calretinin in 20% [9]. However, a minority of patients show no immunohistochemical pattern, as seen in this patient with negative results for CK7 and calretinin [4]. The differential diagnosis for spindle cell carcinoma primarily involves distinguishing it from other conditions that can present with spindle-shaped cells. Immunohistochemistry is an essential diagnostic tool when making this distinction. Among these conditions is the intrapulmonary solitary fibrous tumor, which is usually positive for CD34, BCL2, CD99, and, more recently, STAT6. Inflammatory myofibroblastic tumor is another type of cancer that typically shows spindle cells positive for α-SMA. Similarly, pulmonary myxoid sarcoma presents these cells under the microscope alongside myxoid stroma; however, no immunohistochemical markers have been associated with its diagnosis [10]. Other considerations to take into account would include whether the tumor has a metastatic origin. However, since this is a primary disease without evidence of metastasis, this possibility is ruled out. On the other hand, similarly to this case, a study by Zhang et al. confirmed that PSCC has a predilection for developing in the upper lobe of the lung, with 52.1% of the cases in their study located in that area [2]. Therefore, the tumor location, the cellular morphology observed in microscopy, and the negative results for markers such as S100, SOX10, CD45, and C35 further guide the PSCC diagnosis and rule out other cellular origins of malignancy.

This patient’s case is important to consider due to its genetic medical history. NF1 is a genetic disorder inherited in an autosomal dominant pattern, typically presenting as a benign condition, though malignant transformations are uncommon. The types of cancer most frequently associated with malignant transformation in neurofibromatosis type 1 include chondrosarcoma, osteosarcoma, angiosarcoma, malignant peripheral nerve sheath tumors, and rhabdomyosarcoma [11]. Developing a PSCC in conjunction with NF1 is already rare, so establishing a strong relationship between these two conditions would be an interesting topic for future research.

It is important to emphasize the correct histological interpretation and tissue sampling for these types of lesions, as the histological subtype is often overlooked and misdiagnosed [2]. This case was no exception, as a second biopsy was necessary to correctly assess the cellular differentiation of the tissue. According to established literature, the prognosis for this type of malignancy is poor, with a survival rate of 10% at two years post-diagnosis [12]. Since no specific treatment exists for these lesions, there is no data establishing one chemotherapy regimen as superior to another, which underscores the need for more prospective studies in search of better therapeutic options targeting the improvement of PSCC prognosis.

## Conclusions

Spindle cell carcinomas of the lung are rare and aggressive neoplasms that typically present in male patients over the age of 60. However, this case presents a young woman without any known risk factors, deviating from the expected presentation. The patient’s age together with the use of a combined chemotherapy may be related to the unexpected, prolonged duration of her unresectable Stage IV disease, despite the poor prognosis. This result suggests the need to further review and expand the existing literature, especially regarding the treatment approach.
